# Genome Editing Using Mammalian Haploid Cells

**DOI:** 10.3390/ijms161023604

**Published:** 2015-10-01

**Authors:** Takuro Horii, Izuho Hatada

**Affiliations:** Laboratory of Genome Science, Biosignal Genome Resource Center, Institute for Molecular and Cellular Regulation, Gunma University, 3-39-15 Showa-machi, Maebashi, Gunma 371-8512, Japan; E-Mail: horii@gunma-u.ac.jp

**Keywords:** haploid, embryonic stem cell, CRISPR/Cas

## Abstract

Haploid cells are useful for studying gene functions because disruption of a single allele can cause loss-of-function phenotypes. Recent success in generating haploid embryonic stem cells (ESCs) in mice, rats, and monkeys provides a new platform for simple genetic manipulation of the mammalian genome. Use of haploid ESCs enhances the genome-editing potential of the CRISPR/Cas system. For example, CRISPR/Cas was used in haploid ESCs to generate multiple knockouts and large deletions at high efficiency. In addition, genome-wide screening is facilitated by haploid cell lines containing gene knockout libraries.

## 1. Introduction

**Figure 1:**
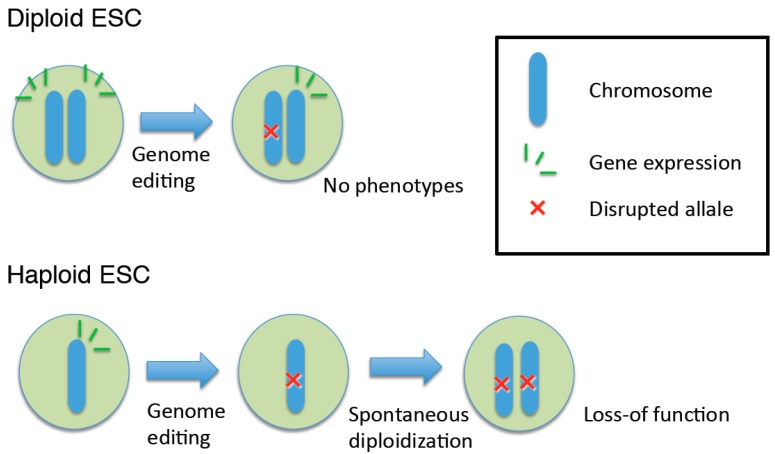
Haploid embryonic stem cells (ESCs) are useful for studying gene functions because disruption of a single allele can cause loss-of-function phenotypes.

Ploidy refers to the number of sets of chromosomes in a cell. Diploid, meaning two chromosome sets, is the most common ploidy in animals. Diploid animals generally produce haploid gametes: for example, a mouse (*M. musculus*) has two genome sets (2*n* = 40) in somatic cells, and generates haploid (*n* = 20) sperm and oocytes. However, these haploid cells are generally limited in their life cycle. For genetic analysis, haploid cells have several advantages over diploid cells for the study of gene function. In diploid cells, heterozygous mutations often lead to no phenotypic changes because a functional allele on a second chromosome set can mask the effects of the disruption of the same allele on the first chromosome set (Figure 1). Genetic analysis in diploid cells is therefore complex, and before genome editing technologies, such as CRISPR/Cas, became available, the analyses had to be performed either by selecting “targeted” clones undergoing loss-of-heterozygosity or sequentially targeting both chromosomes using different resistance genes [1,2]. On the other hand, haploid cells contain only one copy of each chromosome, and disruption of one allele can therefore produce a loss-of-function phenotype (Figure 1). Therefore, gene manipulation in haploid cells can simplify genetic analysis and provide valuable information regarding the molecular mechanisms of numerous genes and proteins. However, these haploid cells are generally limited in their life cycle.

In vertebrates, the first haploid cell lines were obtained more than 40 years ago from frogs [3]. Despite this, the availability of isolated vertebrate haploid cell lines is limited. Mammalian (especially human) haploid cell lines are of particular interest, because genetic screening in mammalian cells is directly applicable to medicinal and pharmacological research. Since the 1970s, several strategies have been used in attempts to produce murine haploid embryos [4,5,6,7]. Successful generation of diploid ESCs in mice [8] suggested that production of haploid ESC lines would be possible; however, the haploid cells became spontaneously diploid during ESC establishment [9]. Haploid ESC lines were recently produced from medaka fish [10]. This led to renewed attempts to generate mammalian haploid ESCs, and mouse haploid ESCs were subsequently produced by two different research groups [11,12].

## 2. Derivation of Haploid ESCs

**Figure 2:**
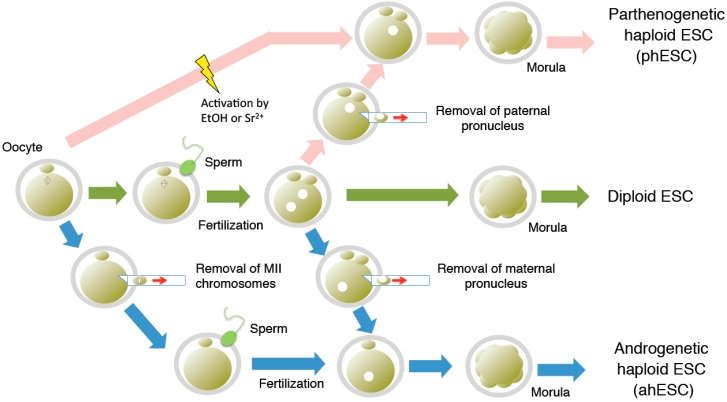
Generation of haploid ESCs via a range of methods. Pink arrows: generation of phESC; blue arrows: generation of ahESC; green arrows: generation of normal diploid ESC; yellow shape: artificial activation by ethanol (EtOH) or strontium chloride (Sr^2+^).

The success in generating mouse haploid ESCs was due to advances in ESC culture systems and improvements in the purification of haploid cells using fluorescence-activated cell sorting (FACS). The first mammalian haploid ESCs were generated from parthenogenetic embryos produced by artificial activation of oocytes with ethanol [11] or strontium chloride [12] (Figure 2). Subsequently, androgenetic haploid ESCs were generated from androgenetic embryos containing only the paternal genome set [13,14]. Androgenetic embryos were produced by removal of the maternal pronucleus [14] or by sperm injection into enucleated oocytes [13,14] (Figure 2). Both parthenogenetic and androgenetic haploid ESCs were functional in chimeric mice, and were able to differentiate to several tissues *in vitro* [11,12,13,14]. Remarkably, the haploid cells underwent diploidization during the differentiation process both *in vivo* and *in vitro* (Figure 1). Genomic imprinting, an epigenetic phenomenon via which certain genes are expressed in a parental origin-specific manner, was partially retained in both androgenetic and parthenogenetic haploid ESCs. Androgenetic and parthogenetic ESCs could therefore be substituted for sperm [13,14] and oocytes [15], respectively. Complete haploid ESCs were generated in other species: for example, androgenetic haploid ESCs were generated in rats [16] and parthenogenetic haploid ESCs in monkeys [17].

By contrast, near-haploid cell lines have been generated from human tumors [18] and used in genetic screens to screen for human host factors involved in infection by pathogens [19,20,21] and the toxic effects of bacterial toxins [22,23,24]. This work has laid the foundation of haploid genetics in mammals today. Human haploid ESCs have not yet been reported; however, successful generation of non-primate haploid ESCs suggests that generation of human haploid ESCs may be possible in the near future.

## 3. Purification and Maintenance of Haploid ESCs

**Figure 3:**
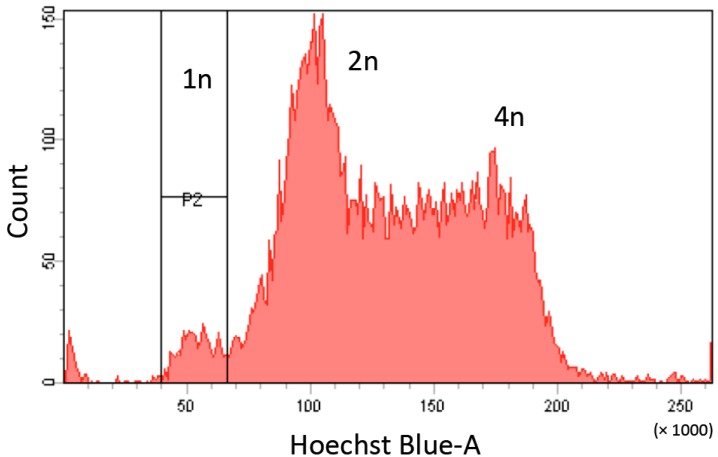
Identification of haploid ESCs by Hoechst 33342 staining. Cells with haploid G1 content (1*n* peak) are purified by fluorescence-activated cell sorting (FACS) using a near-UV laser (375 nm).

Haploid ESCs tend to diploidize spontaneously due to endoreduplication or missed cytokinesis [25]. Haploid ESCs generally become completely diploid within 3 weeks. It is therefore necessary to purify cells with haploid G1 DNA content at certain intervals (Figure 3). Generally, haploid cells are purified by cell sorting after staining with Hoechst 33342, which is detected using a 375 nm near-ultra-violet (UV) laser. However, as most FACS equipment does not have near-UV detection, improved sorting techniques are needed to increase access to haploid ESC research.

**Figure 4:**
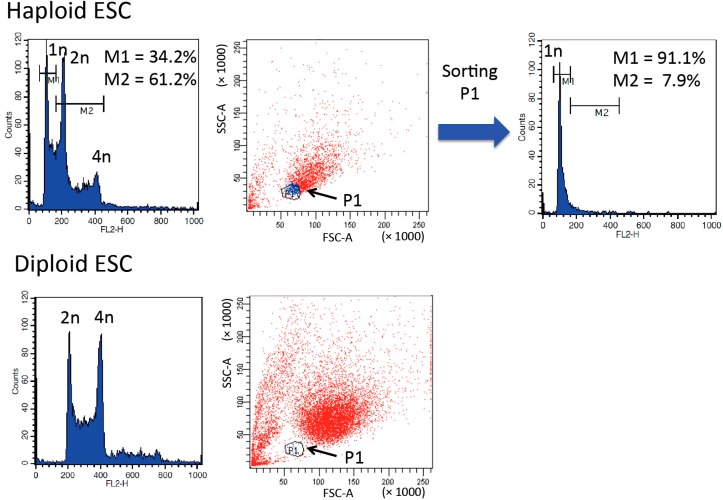
Purification of 1n peak of haploid ESCs using a forward scatter (FSC)/side scatter (SSC) scatter plot to determine cell size. Propidium iodide staining after fixation confirmed that population 1 (P1) contained haploid G1 ESCs and that the P1 population was largely absent in diploid ESCs.

Recently, we and others [26] developed a novel haploid purification system that used a scatter plot generated by FACS analysis with a standard 488 nm laser. No cell staining was required in this system. Scatter plots show forward scatter (FSC) and side scatter (SSC) and can be used to distinguish the physical properties of a particle, such as size and internal complexity. At G1 phase, haploid ESCs are smaller than diploid ESCs (10.0 ± 1.0 µm *vs.* 16.5 ± 2.2 µm in diameter). The novel purification method was able to distinguish haploid ESCs, as indicated by the circled population (P1, blue dots) in the scatter plot in Figure 4. Haploid G1 DNA content was confirmed in the P1 population after cell sorting. In this purification system, the most important procedure is selecting a P1 population, which is largely absent from diploid cells. Therefore, it is essential to use diploid ESCs as a negative control.

Accurate cell sorting is just one requirement for successful maintenance of a haploid ESC population. It is also important to inhibit spontaneous diploidization during daily culture of haploid ESCs. Glycogen synthase kinase (GSK) 3 and ERK inhibitors (2i) are important inhibitors of haploid ESC diploidization [25]. Stability of the haploid karyotype is increased in the naïve ground state by using 2i-supplemented ESC medium. By contrast, exit from the naïve ground state induces diploidization. For example, addition of FGF/activin to growth medium accelerates diploidization by prompting a shift from the naïve ground state to the primed state in epiblast stem cells (EpiSCs) [25]. Dosage compensation occurs in female XX diploid EpiSCs via Xist expression and heterochromatic inactivation of one X chromosome [27]. In these diploid cells, the ratio of X-linked (X) to autosomal gene expression (A) is 1:2. In haploid cells, the gene expression ratio of X:A is 1:1. Haploid EpiSCs, with abnormal dosage compensation might be unstable for cell survival and stimulate diploidization. Inclusion of 2i in growth medium is thus key to inhibiting diploidization of haploid ESCs.

Takahashi and colleagues suggested that diploidization occurred partly as a result of abnormal cell cycle regulation in haploid cells. In this process, cell division was skipped once and cells entered an extra G1/S phase [28]. To overcome this spontaneous diploid conversion, researchers applied culture medium containing a small molecular inhibitor of Wee1 kinase. This regulated the cell cycle by accelerating the G2/M phase transition and preventing entry into the extra G1/S phase. Haploid status was maintained under these conditions, without cell sorting by FACS, for at least 4 weeks.

## 4. Genome Editing in Haploid ESCs

Haploid ESCs are valuable tools for reverse and forward genetic screens. For example, Elling and colleagues used retroviral mutagenesis to generate insertional mutants in haploid ESCs [11]. A forward genetic screen in haploid ESCs by the same authors identified Gpr107 as a key molecule involved in ricin toxicity. Such experiments might be streamlined by the application of recently-developed genome-editing techniques.

The clustered regularly interspaced short palindromic repeats (CRISPR) system, which uses the RNA-guided nuclease Cas9, is a novel genome-editing technology for mammalian cells [29,30,31,32]. This system uses a single gene encoding the Cas9 protein alongside a small guide RNA (sgRNA) complementary to the target DNA sequence [33]. The Cas9 endonuclease generates sequence-specific double-strand breaks (DSBs) at target DNAs bound to sgRNAs. The subsequent repair of the break by non-homologous end joining (NHEJ) frequently introduces mutations. The CRISPR/Cas system has been used to perform high-efficiency loss-of-function screening in mouse and human diploid cell lines [34,35,36]. It is also effective for gene knockout and knockin in human induced pluripotent stem cells (iPSCs) [32], suggesting that it could be used to generate disease model cell lines [37,38] and obtain precise correction of mutant genes [39,40,41,42,43] in human iPSCs.

**Figure 5:**
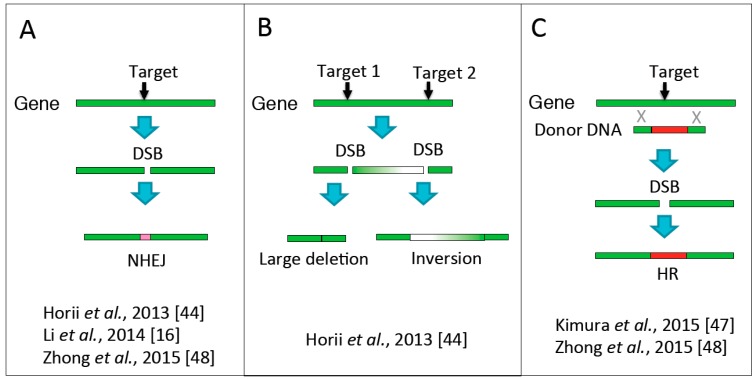
Genome editing in haploid ESCs using the clustered regularly interspaced short palindromic repeats (CRISPR)/Cas system. (**A**) Gene disruption by induction of double-stranded-break (DSB) formation followed by repair by non-homologous end joining (NHEJ); (**B**) deletion or inversion of large genomic regions as a result of two induced DSBs; (**C**) gene knock-in by homologous recombination (HR) with donor DNA.

Recently, we reported the use of the CRISPR/Cas system to manipulate mammalian genes in mouse haploid ESCs [44]. Haploid ESCs were co-transfected with vectors expressing Cas9 nuclease and sgRNAs targeting the *Tet1*, *Tet2*, and *Tet3* genes. Simultaneous disruption of all three genes and corresponding loss-of-function was observed at high frequency (10/20 clones, 50%) (Figure 5A). This targeting (triple knockout) efficiency in haploid ESCs is higher than that previously reported in diploid ESCs (20/96 clones, 20.8%) [45] and in our previous unpublished study (2/20 clones, 10%). Similar experiments using rat haploid ESCs [16] also mutated all three *Tet* genes with high efficiency (3/22 clones, 13.6%). Large chromosome deletions can be produced by using a pair of sgRNAs. Canver and colleagues reported that cleavage of genome sequences can result in the removal of 1 Mb of DNA from mammalian diploid cells [46]. For example, the frequency of biallelic large deletions was 2.5% (2/80 clones) for a 13.5 kb deletion, and 10.1% (16/158 clones) for a 15 kb deletion. In our study, haploid ESCs were also co-transfected with vectors expressing Cas9 and sgRNAs targeting two loci (*Tet1* exons 4 and 7) [44]. This led to the deletion of a 14 kb chromosomal fragment (6/20 clones, 30%) or inversion of the large fragment (2/20 clones, 10%) in a substantial proportion of transfected cells (Figure 5B). Successful CRISPR/Cas-mediated knock-in was also reported in mouse haploid ESCs [47,48] (Figure 5C). Most recently, Zhong and colleagues conducted CRISPR/Cas-mediated genetic screening in mouse haploid ESCs carrying a guide RNA library [48]. Genome-edited mouse androgenetic haploid ESCs were used that could support full-term development of semi-cloned embryos upon injection into MII oocytes. The CRISPR/Cas system is therefore especially powerful for manipulation of mammalian genomes when performed in haploid ESCs. In particular, this will facilitate reverse and forward genetic screens.

We have to discuss potential off-target effects in haploid cells. The off-target effect involves non-specific recognition and digestion at non-targeted regions by the CRISPR/Cas system. Haploid cells contain only one copy of each chromosome; thus, disruption of one allele can lead easily to a loss-of-function phenotype. However, this is a double-edged sword because disruption of one allele on off-target site(s) can produce undesirable loss-of-function phenotypes. In several human tumor cell lines, a high frequency of off-target effects was observed [49,50,51], whereas a low frequency was observed in mouse and its ESC lines [45,52]. However, off-target effects can occur in any cell line. Off-target effects could be reduced in haploid cells by employing the double-nicking strategy with Cas9 nickase, which cleaves only single stranded DNA, and a pair of guide RNAs [53].

The major advantage of using haploid cells is the ease with which genomic manipulation and observation of phenotypes can be performed. Another attractive feature is that ESCs can be maintained as haploid cells only for the duration of editing and then allowed to become diploid (and homozygous for the introduced mutation) for further characterization. In addition, haploid ESCs exhibit pluripotency or multipotency; therefore, screened ESCs can be differentiated both *in vivo* and *in vitro*. Near-haploid tumor cell lines that have been used for haploid genetics in human do not have this feature. If human haploid ESCs are generated in the near future, tissues and organs derived from the screened ESC lines could be used for various analyses.

## 5. Conclusions

Use of the CRISPR/Cas genome-editing system in haploid ESCs allows efficient genetic manipulation of the mammalian genome. This technique provides a new platform for genetic analyses of complex biological phenomena and diseases.
